# Effects of a six-week period of congested match play on plasma volume variations, hematological parameters, training workload and physical fitness in elite soccer players

**DOI:** 10.1371/journal.pone.0219692

**Published:** 2019-07-25

**Authors:** Karim Saidi, Hassane Zouhal, Fatma Rhibi, Jed M. Tijani, Daniel Boullosa, Amel Chebbi, Anthony C. Hackney, Urs Granacher, Benoit Bideau, Abderraouf Ben Abderrahman

**Affiliations:** 1 Movement, Sport, Health and Sciences Laboratory (M2S), University of Rennes 2, Rennes, France; 2 Higher Institute of Sport and Physical Education of Ksar-Said, University of Manouba, Tunis, Tunisia; 3 Laboratory of Biomonitoring of the Environment, Faculty of Science of Bizerte, University of Carthage, Bizerte, Tunisia; 4 Catholic University of Brasilia, Taguatinga, Brazil; 5 Faculty of Medicine of Tunis, University of Tunis, Tunis, Tunisia; 6 Department of Exercise & Sport Science, University of North Carolina, Chapel Hill, NC, United States of America; 7 Division of Training and Movement Sciences, University of Potsdam, Potsdam, Germany; Instituto Politecnico de Viana do Castelo, PORTUGAL

## Abstract

**Objectives:**

The aims of this study were to investigate the effects of a six-week in-season period of soccer training and games (congested period) on plasma volume variations (PV), hematological parameters, and physical fitness in elite players. In addition, we analyzed relationships between training load, hematological parameters and players’ physical fitness.

**Methods:**

Eighteen elite players were evaluated before (T1) and after (T2) a six-week in-season period interspersed with 10 soccer matches. At T1 and T2, players performed the Yo-Yo intermittent recovery test level 1 (YYIR1), the repeated shuttle sprint ability test (RSSA), the countermovement jump test (CMJ), and the squat jump test (SJ). In addition, PV and hematological parameters (erythrocytes [M/mm^3^], hematocrit [%], hemoglobin [g/dl], mean corpuscular volume [fl], mean corpuscular hemoglobin content [pg], and mean hemoglobin concentration [%]) were assessed. Daily ratings of perceived exertion (RPE) were monitored in order to quantify the internal training load.

**Results:**

From T1 to T2, significant performance declines were found for the YYIR1 (p<0.001, effect size [ES] = 0.5), RSSA (p<0.01, ES = 0.6) and SJ tests (p< 0.046, ES = 0.7). However, no significant changes were found for the CMJ (p = 0.86, ES = 0.1). Post-exercise, RSSA blood lactate (p<0.012, ES = 0.2) and PV (p<0.01, ES = 0.7) increased significantly from T1 to T2. A significant decrease was found from T1 to T2 for the erythrocyte value (p<0.002, ES = 0.5) and the hemoglobin concentration (p<0.018, ES = 0.8). The hematocrit percentage rate was also significantly lower (p<0.001, ES = 0.6) at T2. The mean corpuscular volume, mean corpuscular hemoglobin content and the mean hemoglobin content values were not statistically different from T1 to T2. No significant relationships were detected between training load parameters and percentage changes of hematological parameters. However, a significant relationship was observed between training load and changes in RSSA performance (r = -0.60; p<0.003).

**Conclusions:**

An intensive period of “congested match play” over 6 weeks significantly compromised players’ physical fitness. These changes were not related to hematological parameters, even though significant alterations were detected for selected measures.

## Introduction

In contemporary professional soccer, teams are confronted with a large number of matches throughout their competitive season, including league, cup, and international matches [1]. Tight timetables frequently demand that teams play consecutive matches with less than three to four days of recovery in-between competitions. It has been recommended that the recovery period between matches should be at least 72 h to avoid injuries and excessive residual fatigue [2]. Previous studies examined the effects of such a “congested period” of match play on physical and physiological performances in elite soccer players [3] and demonstrated that running activity wasnegatively affected during such periods. Of note, skill-related performance can also be affected by a decline in soccer players running capacity [4,5]. These studies provide important insight into the demands of congested match play, but they are limited in scope and few in number. For the safety of players, more research is warranted to better examine the negative effects of different congested periods on physical fitness, technical and tactical performance in elite soccer players. Furthermore, little is known on how an intensified in-season soccer period affects critical physiological markers such as hematological parameters and how these parameters may affect and explain the impairment of physical performance during congested periods of match play in soccer players.

Excessive physical exercise may influence body homeostasis and may result in impaired immune function and changes in hematological variables. This could lead to a state of overreaching which ultimately affects physical performance. It is mandatory [6] to monitor players’ physical fitness together with physiological markers during different periods of a soccer season to improve training quality and to promote health for instance through lowering the risk of sustaining injuries. To this end, blood samples could be taken to monitor changes in hematological variables (e.g., plasma volume, hematocrit, hemoglobin) [6].

It is well known that most common hematological measurements (e.g., plasma volume [PV], hemoglobin [Hb], hematocrit [Ht], and red blood cells [RBC] count) are linked to the development of aerobic capacity [7]. Previous research suggested that intensive physical stress affects PV and hematological parameters, which may negatively influence players’ physical performance [8,9]. Variation in PV and the parameters associated with it is considered an important form of body fluid adaptation in response to different training loads [10] and exercise intensity [11]. For example, Hb values determine the oxygen transport and consumption [12], which is linked to physical performance through aerobic capacity [13]. Ht levels are usually associated with enhancements in oxygen transport capacity [14]. The main function of RBC is oxygen transport and consumption. Thus, the longitudinal monitoring of Hb, Ht and RBC over 6 months revealed increases in hematological parameters at the end of the pre-season period in professional football [6]. According to Silva et al. [15], Ht was significantly increased after 3 months of training while it was decreased during the more intense periods of training in Brazilian soccer players. Moreover, Heisterberg et al. [16] monitored these hematological parameters in players from the best Danish league, and found a decrease in Hb and Ht as the competitive season progressed. In addition, the highest value of Ht and the lowest value of Hb were obtained after a period characterized by frequent matches [16]. Hence, these Blood parameter variations are related to the amount of aerobic and anaerobic training, strength training, and the number of matches per week [16]. Given the importance of these measurements for physical fitness, it is imperative to better understand the concurrent effects of a congested period of matches on physical and hematological parameters for a better management of training and competitive workloads.

To our knowledge, no studies have examined PV and hematological parameters of elite soccer players from the same team, before and after a congested period of match play and training. In addition, there are no studies available that quantified the relationships between training load parameters, hematological parameters and physical fitness. Thus, the aims of this study were twofold: i) to investigate the effects of a congested fixture period on PV, hematological parameters, and physical fitness in elite soccer players; and ii) to examine the relationships between training load, hematological parameters and physical fitness of elite soccer players. It was hypothesized that successive matches played during a short time period could negatively affect PV, hematological parameters which may have an impact on players’ physical fitness [1, 16].

## Materials and methods

### Participants

Eighteen elite male Tunisian soccer players with a mean age of 20.1±0.4 years from the same first division Tunisian football team volunteered to participate in this study. Participants included three central backs, four fullbacks, seven midfielders, and four attackers. All players had been engaged in systematic training programs and in national competitions for at least 10 years. The included players were those with the longest match time (i.e., players who were most often in the starting line-up). Thirteen players performed all matches. The other 5 players trained with the first team and played their matches with the reserve team. The first and the reserve team had the same match schedule. None of the participants reported recent injuries that could interfere with study participation. Each player provided written informed consent to participate in this study after being fully informed about all experimental procedures. The study was carried out in accordance with the guidelines contained in the Declaration of Helsinki and was approved by the Ethics Committee of the medical unit of the included soccer club (Jeunesse Sportive Kairouanaise, JSK) and the Ethics Committee of the Scientific Council of the University of Rennes 2, France.

### Procedures

**Fig 1:**
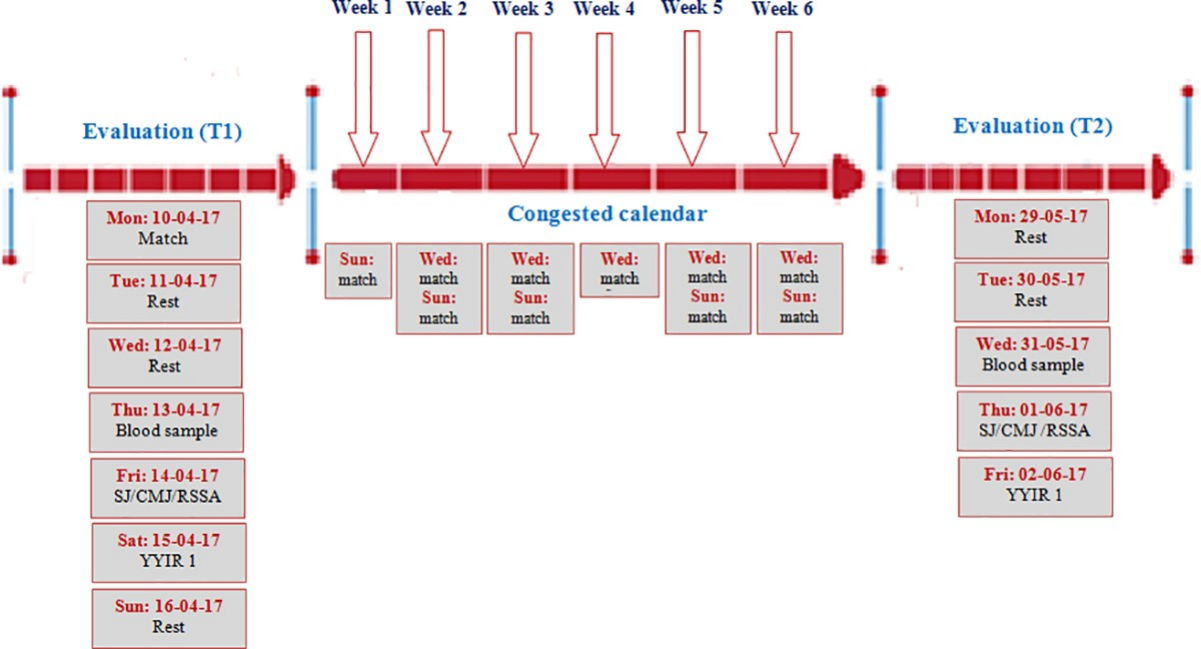
Experimental design. Evaluation T1: Before the period of match congestion Evaluation T2: After the period of match congestion Congested calendar: A six-week period of congested match play Mon: Monday, Tue: Tuesday, Wed: Wednesday, Thu: Thursday, Fri: Friday, Sat: Saturday, Sun: Sunday, Rest: a day of recovery RSSA: Repeated sprint shuttle ability SJ: Squat jump CMJ: countermovement jump YYIR: Yoyo intermittent recovery.

All players were evaluated before and after a period of congested match play during the competitive season’s play-off period. The first test session (T1) took place in the middle of April, while the second test session (T2) was performed at the end of the season (late May until early June), after 6 weeks of competition. Evaluation of hematological parameters and physical fitness were performed over three-days. On the first day, a blood sample was collected to determine resting PV and hematological parameters. On the second day, participants performed three physical fitness tests in the following sequence: squat jump test (SJ), countermovement jump test (CMJ), and repeated shuttle sprint ability test (RSSA). On the third day, participants performed the Yo-Yo intermittent recovery test level 1 (YYIR1) (see **Fig 1** for experimental design). All tests were performed in the afternoon, 3 hours after having taken a light standardized meal. In general, the applied physical fitness tests chosen were regularly employed to monitor and assess players’ fitness over the course of the season. To minimize diet-related changes in performance, players were asked to standardize and follow the same nutritional plan 24 hours before each testing session. Daily ratings of perceived exertion (RPE; Borg scale) were assessed in order to quantify the internal training load [17].

During the 6-weeks period, 10 matches were played. The number and duration of training sessions were the same for all players. The overall number of sessions was 26 (Table 1). The duration of each training session was 1 h 30 min for all players. Ten games were played over the 6-weeks study period. Training sessions were performed during the morning hours.

**Table 1:** Training program during the congested period.

Day	Weekly program when playing one match per week (weeks 1 and 4)	Weekly program when playing two matches per week (weeks 2, 3, 5, and 6)
**Monday**	Recovery	Warm up, 15 min Low-to-moderate intensity aerobic training Tactical training, 30 min Technical training, 35 min
**Tuesday**	Warm up, 15 min Technical training, 30 min Low-to-moderate intensity aerobic training, 25 min Small sided games, 15 min	Warm up, 15 min Tactical training, 30 min High-intensity aerobic training, 10 min Small sided games, 15 min
**Wednesday**	Warm up, 15 min Strength training, 30 min Technical tactical training, 20 min Reduced game, 15 min	Match
**Thursday**	Warm up, 15 min Tactical training, 35 min Speed training, (short distances), 20 min Small sided games, 15 min	Low-to-moderate intensity aerobic training, 40 min Soccer specific training, 30 min
**Friday**	Warm up, 15 min Technical training, 30 min Speed training (long), 20 min Soccer specific training, 20 min	Warm up/ technical, 25 min Speed training (short), 15 min Small sided games, 25 min
**Saturday**	Warm up /technical, 25 min Speed training (short), 20 min Soccer specific training, 30 min	Recovery
**Sunday**	Match	Match

The 1^st^ week and the 4^th^ week were scheduled with 5 sessions per week, which were conducted on Tuesday, Wednesday, Thursday, Friday, and Saturday. On the 2^nd^, 3^rd^, 5^th^, and 6^th^ week, four training sessions per week were executed on Monday, Tuesday, Thursday, and Friday. In addition, two matches were played per week (Wednesday and Sunday). Full recovery (i.e., day off) was provided on Saturday (see Table 1). The duration of each training session was 1h 30 min for all players. The weekly training load was evaluated from Monday to Sunday. The training program consisted of low/moderate intensity continuous running (i.e., 50–70% of maximum heart rate [HRmax]), high-intensity interval running (i.e., 90–100% of HRmax), specific soccer training drills according to the respective playing position, speed ‘sprint’ training over 10-30-m (short) and 30-60-m (long) with and without a ball, tactical training, technical training (attack against defense in small fields), small sided games, and strength training.

The strength training during the congested period was different with regards to duration and intensity from the pre-season or competitive season. The applied strength training sessions (25 min each week) consisted of circuit training to primarily improve muscle power. Plyometric exercises together with high velocity strength exercises were performed during circuit training. After strength training, players conducted shots to the goal. All training sessions were preceded by a 5–15 min standardized warm-up. All players completed all training sessions and completed all matches. However, only 3 substitute players had less match time than the rest of the cohort. Therefore, training load of the substitution players was increased during the day after the match to reach a similar overall level as the players who were active during the match. Table 1 summarizes the training characteristics during the congested period of match play.

### Anthropometric testing

Body mass (kg) was measured to the nearest 0.1 (kg), with the participant in light clothing and without shoes, using a mechanical scale (M-i525, medical robe, France). Body height (m) was determined to the nearest 0.5 cm using a measuring tape fixed to a wall. The body mass index (BMI) was subsequently calculated (kg/m^2^). Percent body fat (%BF) was estimated using four skin folds (biceps, triceps, sub-scapular, and suprailiac) measurements and assessed with a skin fold caliper (CZ-store, France) [18]. Lean body mass was calculated using the equation of Forsyth et al. [19], LBM (kg) = mass—(BF [%] × weight / 100).

### Blood analyses

For the hematological parameters, 15 ml of venous blood were collected in the morning between 08:00–10:00 am. Blood samples were collected following an overnight fast, at least 12 h without exercise, and after the participants had remained seated for 30 min. Blood samples were drawn via the median ante brachial vein into vacutainer tubes (Vacuette, Greiner bioOne, France) without anti-coagulant and with EDTA. After being centrifuged at 2,500 (rpm) for 10 (min), the serum was stored at -70° C for determination of hematological parameters; erythrocytes (M/mm^3^), hematocrit (%), hemoglobin (g/dl), mean corpuscular volume (fl), mean corpuscular hemoglobin content (pg), and mean hemoglobin concentration (%), in a flow cytometry (CelltacES, Japan).

The measured Ht and Hb were utilized in order to calculate plasma volume changes (ΔPV) and expressed as % ΔPV [20].

$$\%\Delta PV=100\times [\frac{Hb_{B}}{Hb_{A}}\times \frac{\left(1-Ht_{A}\times 10^{-2}\right)}{\left(1-Ht_{B}\times 10^{-2}\right)}]-100$$

Where B refers to resting values (before the period of match congestion) for Ht and Hb, and A refers to the subsequent values for Ht and Hb after the period of match congestion.

### Yo-Yo intermittent recovery test level 1 (YYIRTL1)

The YYIRT1 was used to assess players’ endurance performance and the ability to repeat high-intensity exercise. Cones were placed on 2 lines 20 m apart and 5 m behind the starting line. Players ran two 20 m shuttles on a beep signal, separated by a 10 s recovery. The beep increased in stages and players continued the test until they were unable to maintain the required pace for 2 successive beeps. Players were required to place 1 foot either on or behind the 20-m line at the end of each shuttle. If a player did not reach the line in time, he was instructed that he must do so on the following run. The final stage (km/h) achieved and total distances covered (m) were recorded.

### Repeated shuttle sprint ability

Repeated shuttle sprint ability (RSSA) was used to assess players’ ability to cope with the intermittent demands of soccer. This test includes rapid changes of direction, which represents sport-specific movement patterns in soccer.

After a 15 min warm up, players completed 6 × 40 m maximal sprints interspersed with 20 s of passive recovery. Sprint times were recorded with a chronometer. The best sprint time was recorded for further analysis (RSSA_best_). In addition, dependent variables comprised mean time obtained over six sprints, and the decrement in performance (RSSA_decrement_ = ([RSA_mean_] / [RSA_best_] × 100)– 100) [21]. Blood lactate concentration was determined at the end of the test using a portable lactate analyzer (Lactate Pro2, Matsport, France).

### Squat jump (SJ) and countermovement jump (CMJ)

The squat jump (SJ) started from a static semi-squatting position with knees at approximately ~90°, and, after a short rest (1 s), the player jumped at maximal effort as high as possible. For the countermovement jump (CMJ), players started in a standing position, and performed an explosive jump using a slow stretch-shortening cycle (SSC) at ~90° knee flexion. Players were asked to jump at maximal effort and as high as possible. All players performed their vertical jumps in the same order (i.e., SJ followed by CMJ) with hands akimbo. All players performed three trials for each jump test, with a 60 s interval between each trial. The mean of the best two trials was taken for further analysis [22].

### Quantification of training load

The total weekly training load (TWTL) was quantified on a daily basis by means of the session rating of perceived exertion (session-RPE) using an adapted Borg’s 0–10 scale [17]. To ensure that the rating of perceived exertion was reflective of the entire session, data was collected 15–20 min following each training session. Monotony was calculated as the weekly average load divided by the standard deviation of the load. Strain was also calculated as the training load multiplied by monotony [23]. It has previously been suggested that these indices are associated with signs of overreaching and/or overtraining.

### Statistical analyses

Data were expressed as mean values and standard deviations (SD). All statistical tests were conducted using Sigma Stat 3.5 software (Systat, Inc, USA). Normal data distribution was tested using the Kolmogorov-Smirnov test and confirmed. Paired t-tests for dependent samples were used to compare all parameters measured before and after the period of match congestion (T1 vs. T2). A one-way analysis of variance with repeated measures (ANOVA) was computed to determine the difference between the weekly training loads during the period of match congestion. Relationships between parameters were assessed using Pearson’s product-moment correlation coefficients (r). The magnitude of the correlations was defined according to Hopkins’s scale: r<0.1, trivial; 0.1–0.3, small; >0.3–0.5, moderate; >0.5–0.7, large; >0.7–0.9, very large; >0.9, nearly perfect; and 1, perfect. Statistical significance for all analyses was set at p < 0.05. The 95% confidence intervals (CI) and effects sizes (ES) were calculated to compare differences in mean values for all analyzed parameters before and after the six-weeks in-season period. When calculating ES, pooled standard deviations (SD) were used as no control group was available (Cohen’s d = [M1-M2] / SD pooled). ES with values of 0.2, 0.5, and 0.8 were considered small, medium, and large, respectively [24].

## Results

### Anthropometrics

Changes in anthropometrics are illustrated in Table 2. No significant pre-to-post (T1 vs. T2) differences were found for the following measures: body height (p = 0.2; ES = 0.3), body mass (p = 0.1; ES = 0.3), BMI (p = 0.06; ES = 0.4), BF (p = 0.1; ES = 0.1), LBM (p = 0.4; ES = 0.1).

**Table 2:** Effects of a congested period of match play on soccer players’ body height, body mass index (BMI), body fat percentage (BF%), and lean body mass (LBM).

Anthropometric measures	Pre testing (T1)	Post testing (T2)	p-Value	Effect size (ES)
Body height (m)	1.78±0.04	1.78±0.05	0.3	0.2
Body mass (kg)	72.6± 5.1	72.2± ±5.1	0.1	0.3
BMI (kg/m^2^)	22.7±1.19	22.6±1.14	0.06	0.4
BF (%)	8.2±2.9	8.5±2.4	0.1	0.1
LBM (kg)	66.6 ±4.3	66.1± 4.7	0.4	0.1

BMI: Body mass index, BF: Body fat, LBM: Lean body mass.

### Physical fitness

In general, physical fitness declined over the 6-weeks period (Table 3). More specifically, significant decreases were found for the YYIR1 (p≤0.05; ES = 0.5), the RSSA (p≤0.05; ES = 0.6) and the SJ (p≤0.05; ES = 0.5). In contrast, CMJ performance did not change significantly (p = 0.86). Post-exercise blood lactate after the RSSA test increased significantly from T1 to T2 (p = 0.04; ES = 0.5).

**Table 3:** Changes in performance in physical fitness tests after the period of match congestion.

Fitness test	Pre testing (T1)	Post testing (T2)	p-value	Percentage change	Effect size (ES)
YYIR1 (m)	2520±362.6	1640±326.6	<0.001*	34.9%	5
RSSA mean (s)	8.07±0.3	8.28±0.3	0.01*	2.6%	0.6
RSSA best (s)	7.4±0.4	7.7±0.4	0.001*	3.8%	0.7
RSSA decrement (%)	7.8±4.7	6.6±3.1	0.3	0.06%	0.2
[Lac] _RSSA_ (mM/L)	18.2±3.2	20.3±2.3	0.04*	11.5%	0.5
SJ (cm)	36.5±3.8	35.5±3.9	0.01*	2.5%	0.5
CMJ (cm)	39.0±3.6	39.2±2.4	0.8	0.4%	0.1

YYIR1: yoyo intermittent recovery test Level 1, Lac: Blood lactate concentrations; RSSA: repeated sprint shuttle ability, SJ: squat jump, CMJ: countermovement jump.

* = Significant difference between T1 (before the period of match congestion) and T2 (after the period of match congestion).

### Training load

**Fig 2:**
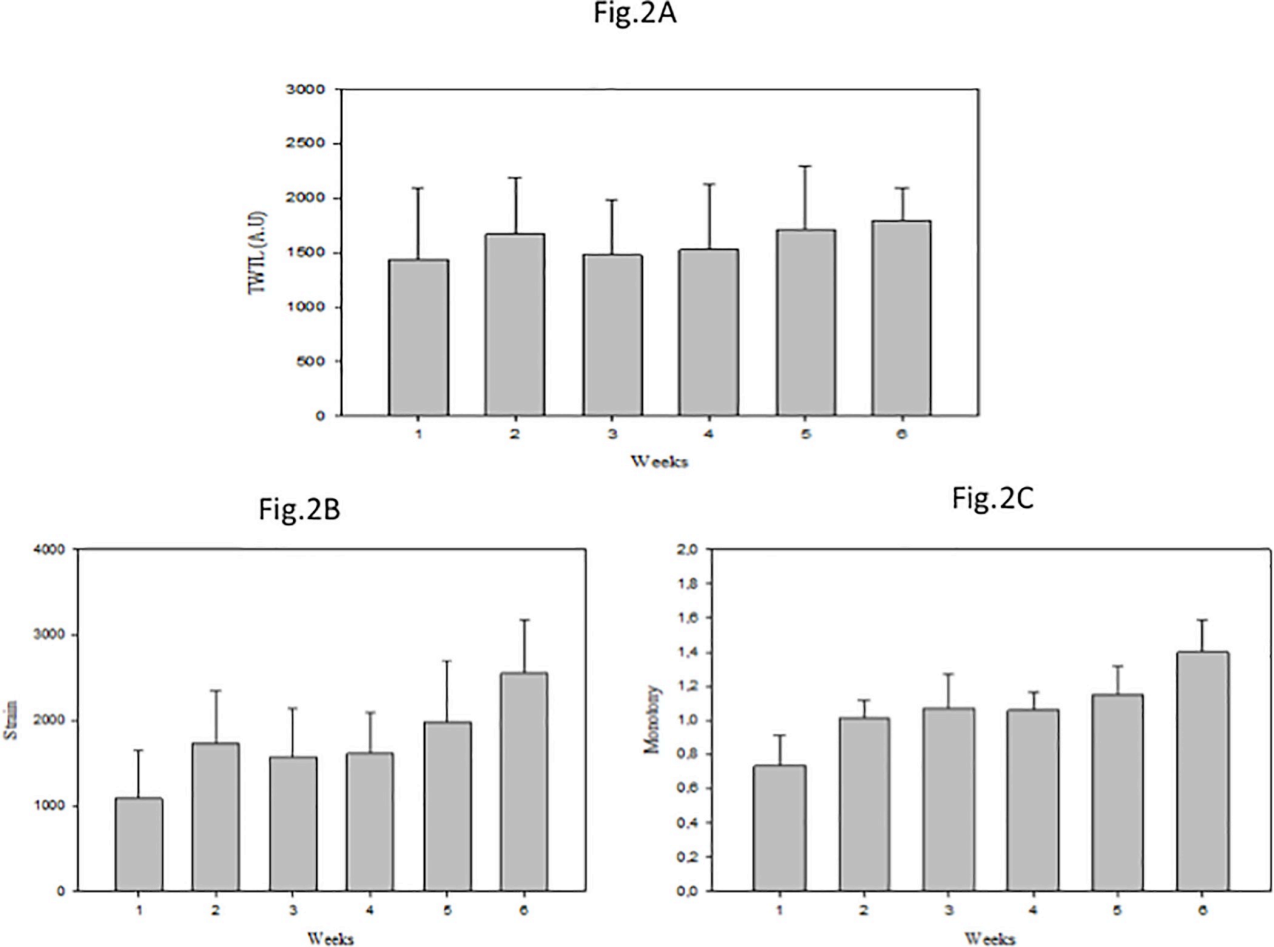
**Total weekly training load (TWTL) (Fig 2A), the monotony (Fig 2B) and the strain (Fig 2C) of the six weeks training during the match congestion period.** ** = Significant differences compared to week 1, week 3, and week 4 (p≤ 0.01). TWTL = Total weekly training load; A.U. = arbitrary units, monotony and strain.

Total weekly training load (TWTL), the calculated training monotony as well as the strain over the six training weeks are illustrated in Fig 2A, 2B and 2C. The TWTL of the 6^th^ week was higher than the TWTL during the first 4 weeks (p≤0.01; see Fig 2A for total weekly training load [TWTL] over the six training weeks of congested match play).

The monotony level of the 6^th^ week was higher than that of the 1^st^ week, the 3^rd^ week, and the 5^th^ week (p≤0.01). The monotony level during the 3^rd^ week, the 4^th^ week, and the 5^th^ week were higher than that during the 1^st^ week (p≤0.01) (see Fig 2B for monotony over the six training weeks of congested match play).

The strain experienced by the players during the 6^th^ week was higher than that during the 1^st^, 2^nd^, 3^rd^, 4^th^, and 5^th^ week (p≤0.01). The strain experienced during the 3^rd^, 4^th^, and 5^th^ week were higher than that during the 1^st^ week (p≤0.01; see Fig 2C for strain over the six training weeks of congested match play).

### Hematological and plasma volume variation

Table 4 summarizes data of all analyzed hematological parameters. A significant decrease was found from T1 to T2 for erythrocyte count (p = 0.018; ES = 0.5), Hb (p = 0.002; ES = 0.8), and Ht percentage (p = 0.006; ES = 0.6). Conversely, MCV, MCHC, and MHC values did not change significantly over the experimental period, while PV increased significantly from T1 to T2 (6.1± 8.7%; p = 0.01; ES = 0.5).

**Table 4:** Effects of the period of match congestion on soccer players’ hematological parameters.

Hematological parameters	Pre-testing (T1)	Post-testing (T2)	p-value	Effect size (ES)
**Erythrocytes (M/mm**^**3**^**)**	5.3 ± 0.4	5.1 ± 0.3	0.018*	0.5
**Hemoglobin (g/dl)**	14.9 ± 1.04	14.3 ± 0.7	0.002*	0.8
**Hematocrit (%)**	43.9 ± 2.7	42.5 ± 2.07	0.0006*	0.6
**MCV (fl)**	83.1 ± 4.5	82.9 ± 4.5	0.1	0.3
**MCHC (pg)**	28.05 ± 1.7	28.09 ± 1.6	0.4	0.1
**MHC (%)**	33.9 ± 0.7	33.7 ± 0.6	0.1	0.3
**PV (T1-T2) (%)**	6.1±8.7%		0.01*	0.7

MCV: Mean corpuscular volume, MCHC: Mean corpuscular hemoglobin content, MHC: Mean hemoglobin concentration, PV (T1-T2): Plasma volume variation between T1 and T2; YYIR1: yoyo intermittent recovery test Level 1, RSSA: repeated sprint shuttle ability, SJ: squat jump, CMJ: countermovement jump.

* = Significant difference between T1 (before the period of match congestion) and T2 (after the period of match congestion).

### Relationships between PV, hematological parameters, physical fitness, and training load parameters

Table 5 shows that there were no significant correlations between PV, Δ% of hematological parameters, and Δ% of physical fitness. However, there were significant positive correlations between Δ% of SJ and Δ% of Ht (r = 0.54; p = 0.02), MCV (r = 0.51; p = 0.01), and MCHC (r = 0.62; p = 0.001). In addition, there was a significant negative correlation between percentage change of SJ and PV (r = -0.43; p = 0.03).

**Table 5:** Relationships (Pearson correlation coefficient) between PV, percentage changes of hematological parameters and percentage change of physical fitness.

Hematological parameters / physical fitness	YYIR1	RSSA mean	SJ
Hemoglobin (g/dl)	p = 0.12; r = 0.21	p = 0.08; r = 0.15	p = 0.07; r = -0.4
Hematocrit (%)	p = 0.11; r = 0.19	p = 0.15; r = 0.21	p = 0.02*; r = 0.54
PV (%)	p = 0.9; r = -0.01	p = 0.4; r = 0.20	p = 0.03* ; r = -0.42
Erythrocytes (M/mm^3^)	p = 0.8; r = 0.04	p = 0.4; r = -0.12	r = 0.4; p = 0.06
MCV (fl)	p = 0.6; r = 0.15	p = 0.3; r = -0.22	p = 0.01*; r = 0.51
MCHC (pg)	p = 0.5; r = -0.12	p = 0.3; r = -0.20	p = 0.001*; r = 0.64
MHC (%)	p = 0.6; r = -0.14	p = 0.2; r = -0.25	p = 0.1; r = 0.32

MCV: Mean corpuscular volume, MCHC: Mean corpuscular hemoglobin content, MHC: Mean hemoglobin concentration, PV (T1-T2): Plasma volume variation between T1 and T2. YYIR1: yoyo intermittent recovery test Level 1, RSSA: repeated sprint shuttle ability, SJ: squat jump, CMJ: countermovement jump.

(*) Significant relationship between parameters.

Table 6 illustrates that there was no significant relationship between training load parameters and Δ% of hematological parameters (Hb [r = 0.12; p = 0.6], Ht [r = -0.02; p = 0.9], PV [r = 0.12; p = 0.6], erythrocytes [r = -0.22; p = 0.2], MCV [r = -0.05; p = 0.9], MCHC [r = 0.22; p = 0.2], MHC [r = 0.41; p = 0.08]). There were no significant relationships between monotony and Δ% of hematological parameters (Hb [r = 0.3; p = 0.1], Ht [r = -0.12; p = 0.6], PV [r = -0.14; p = 0.6], erythrocytes [r = -0.34; p = 0.1], MCV [r = -0.12; p = 0.6], MCHC [r = 0.3; p = 0.1], MHC [r = 0.32; p = 0.1]).

**Table 6:** Relationships (Pearson correlation coefficient) between PV, percentage changes of hematological parameters, physical fitness, and workload parameters.

Hematological parameters and training load	TWTL	Monotony	Strain
Hemoglobin	p = 0.6; r = 0.12	r = 0.30; p = 0.1	p = 0.5; r = 0.14
Hematocrit	p = 0.9; r = -0.02	p = 0.6; r = -0.12	p = 0.8; r = -0.05
PV	p = 0.6; r = 0.12	p = 0.6; r = -0.14	p = 0.6; r = 0.18
Erythrocytes	p = 0.2; r = -0.22	p = 0.1; r = 0.34	p = 0.1; r = -0.34
MCV	p = 0.9; r = -0.05	p = 0.6; r = -0.12	p = 0.7; r = -0.08
MCHC	p = 0.2; r = 0.22	p = 0.1; r = 0.30	p = 0.2; r = 0.30
MHC	p = 0.08; r = 0.41	p = 0.1; r = 0.32	p = 0.08; r = 0.42
YYIR1	p = 0.5; r = 0.14	p = 0.9; r = -0.01	p = 0.6; r = 0.12
RSSA-mean	p = 0.003*; r = -0.60	p = 0.2; r = -0.22	p = 0.005*; r = -0.63
SJ	p = 0.8; r = -0.54	p = 0.5; r = 0.14	p = 0.8; r = -0.03

MCV: Mean corpuscular volume, MCHC: Mean corpuscular hemoglobin content, MHC: Mean hemoglobin concentration, PV (T1-T2): Plasma volume variation between T1 and T2. YYIR1: yoyo intermittent recovery test Level 1, RSSA: repeated sprint shuttle ability, SJ: squat jump, CMJ: countermovement jump, TWTL: Total weekly training load.

(*) Significant relationship between parameters.

Moreover, there were no significant relationships between strain experienced and Δ% of hematological parameters (Hb [r = 0.14; p = 0.5], Ht [r = -0.05; p = 0.8], PV [r = 0.18; p = 0.6], erythrocytes [r = -0.34;p = 0.1], MCV [r = -0.08; p = 0.7], MCHC [r = 0.30; p = 0.2], MHC [r = 0.42; p = 0.08]).

There was no significant relationship between performance in the YYIR1 and training load parameters (r = 0.14; p = 0.5), monotony (r = -0.01; p = 0.9), and strain (r = 0.12; p = 0.6). There was, however, a significant relationship between training load and strain, with RSSA (r = -0.64; p = 0.003; r = -0.63; p = 0.005, respectively).

## Discussion

The main purpose of this study was to analyze the effects of a 6-week period of congested match play on PV, hematological parameters, training load and physical fitness in elite soccer players. In accordance with our research hypothesis, the main results of this study were significant modulations in soccer players’ PV, selected hematological parameters, and physical fitness after an intense 6-weeks match congested period. However, the observed changes were not related to declines in physical fitness other than SJ performance.

In this study, Hb and Ht values decreased after the 6-weeks congested period. It is well established that training may alter homeostasis, including hematological parameters. Hb is a key determinate of oxygen transport and consumption [25], which is related to physical performance, primarily aerobic capacity [26,27]. Elevated Hb is generally associated with an increase in blood oxygen transport capacity and aerobic performance, while an increase in Ht increases blood viscosity [28]. The kinetics of variation of these hematological parameters may partly explain players’ aerobic performance. Thus, it seems beneficial to monitor soccer players’ Hb and Ht parameters.

Several studies showed decreases in Hb and Ht values after periods of intense training or competition [6,7,26]. These declines are known as an adaptation to training and called sports pseudo-anemia [29,6]. In the concept of sports pseudo-anemia, stimulation of the erythrocytosis during exercise induces an increase in the absolute concentration of Hb [6]. This mechanism is masked by a rise in PV [15,30,31]. Sports anemia can be treated as a first sign of overtraining or regular training that can lead to altered aerobic performance [31]. To date, only a few studies have evaluated Hb and Ht variations during a competitive season in soccer players [29, 15]. For example, in a previous study by Silva et al. [15], it was observed that Hb and Ht of Brazilian soccer players were significantly increased after 12 weeks of soccer training. These authors postulated that these alterations occurred due to plasma volume changes (i.e., hemoconcentration). In fact, in this study [15], PV decreased by 11.2% after the soccer-training program and was significantly correlated to the alteration percentage of Hb and Ht changes. In contrast, Heisterberg et al. [16] recorded no significant changes in Hb and Ht levels over a 6-months period in which the training and match load varied considerably. These discrepancies in results may be mainly explained by differences in the experimental protocols involved, and factors such as training intensity, duration, frequency, and players’ expertise level.

In the present study, we hypothesized that the period of congested match play would negatively affect plasma volume and hematological parameters, which, in turn, would lead to negative changes in players’ physical fitness. The current results showed a significant decrease after the period of match congestion with respect to the values of erythrocytes. In agreement with our results, many studies suggest that the number of erythrocytes is decreased at the end of a competitive period in soccer [6]. In general, erythrocytes, Hb, and Ht decrease after endurance training [32]. This is mainly caused by PV expansion [33]. In contrast, Silva and al. [15] showed training-induced increases in erythrocytes after 12 weeks of training in Brazilian soccer players. A possible explanation for this increase might be plasma volume changes [15,26]. In fact, Silva et al. [15] were able to show that the altered percentage of erythrocytes significantly correlated with plasma volume change (i.e., reductions) during the 12-weeks soccer-training program. As to MCV, MHC and MCHC response, our data showed no significant changes after the period of match congestion. These results are not in accordance with findings from Meister et al. [34] who observed a decrease in MCH and MCHC values and an increase in MCV values during a competitive period. These differences in findings can primarily be explained by psychological factors, players’ diet [9,15], and/or differences between players’ effective match time.

In our study, PV measured after a 6-week period of match congestion was significantly higher (+ 6.1%) in T2. In contrast, Silva et al. [15] demonstrated a decrease in PV after 12 weeks of specific soccer training. The specifics of the soccer-training program and/or participants’ expertise level could explain the contradictory findings. For example, our mean weekly training volume was lower (= 9 h/week) than that of a previous study conducted by Silva and colleagues with Brazilian soccer players (14.7–16.3 h/week) [15]. Additionally, our study period lasted 6 weeks which is less than the study of Silva et al. [15] (12 weeks). This might also be a reason for the different outcomes in the two studies.

Previous studies showed that improved endurance performance was accompanied by increases in PV and decreases in Ht [35]. This finding can be explained by auto-hemodilution, which is manifested by the decrease in blood viscosity [36,37]. This decrease in Ht together with the increase in PV improves physical fitness performance by decreasing peripheral vascular resistance and ventricular post load [37]. Thus, the beneficial effects of training were not a decrease in PV and an increase in Ht, but rather the opposite.

Our findings with regards to the observed changes in hematological parameters and how they impacted on performance are contradictory to the results of previous studies [15,35]. In fact, the decline in physical fitness performance of elite soccer player after the period of match congestion was accompanied by an increase in PV and a decrease in the values of Hb and Ht. Thus, our results confirm that the soccer players enrolled in this study did not show signs of sport anemia as was reported for endurance athletes [31]. A likely explanation for the different outcomes could be that training volume of endurance athletes is much higher compared with that of soccer players. The training program of soccer players is characterized by a large variety of training contents (e.g., tactical/technical drills, physical fitness [endurance, strength, speed, power]). Therefore, the training of one specific physical quality is less intense in soccer compared with an endurance related sport that demands high levels of aerobic capacity during training. This may additionally explain the observed discrepancy in outcomes between our study and previous studies. Consequently, the type of training associated with soccer may be the main reason for the observed changes in erythrocytes, Hb, Ht, and PV variations [15,16].

Interestingly, we found no significant correlations between PV, hematological parameters, and players’ performance in the YYIR1 and RSSA tests. In contrast, several studies conducted with endurance athletes and soccer players showed significant negative correlations between Ht and measures of physical fitness, suggesting increased performance with a decreasing Ht value [6,27]. In addition, there is evidence that Ht correlates well with the development of VO_2max_ in young soccer players [38]. Moreover, Abdderrahman et al. [35] showed a significant positive correlation between PV and VO_2max_ in physical education students.

Notably, Silva et al. [38] showed that Ht increased with increasing training volume over a 6-week soccer-training program. This is indicative of the reactivity of hematological parameters to variations in training volume. In addition, Colombini et al. [39] reported that Ht values were higher after a soccer match due to a decrease in PV. In the present study, the changes observed in hematological parameters and training load were not related to declines in physical fitness other than SJ performance. This difference in findings among the studies may in part be explained by experimental factors such as the level of participants’ expertise, the respective training period, the training protocol, and the utilized testing methodology.

The observed decline in players’ physical fitness in this study could mainly be explained by fatigue which occurred after the application of the heavy training load and the large number of matches (10 matches during 6 weeks). Nevertheless, the observed performance decline together with altered hematological markers could also be explained by the acute effects of the last week of competition as opposed to an aggregated effect. In fact, the monotony and strain experienced by our players during the last week were higher compared with that during the previous weeks.

Interestingly, Meister et al. [34] assessed the effects of a 3-week period of high match exposure (>270 min during 3 weeks) compared with a low (<270 min during 3 weeks) match exposure period in German male elite soccer players on hematological markers. In the study of Meister et al. [34], no relevant alterations were observed in the analyzed parameters. It might be argued that the periods of high match exposure were too low to induce alterations in physical fitness. Thus, it seems plausible that the training volume was intentionally reduced during this period to avoid excessive overload [34]. However, the average match exposure in our study was 1.3 matches per week which equals 900 min of match time over the 6 weeks intervention period. This indicates that the high match exposures time of elite soccer players could partly explain the observed performance decline. In contrast to the procedures reported by Meister et al. [34] on training volume, the participating coaches of this study rated training volume as highly important performance criteria which is why similar training volumes were scheduled in weeks with one or two matches.

In this study, physical fitness significantly declined over the 6-weeks experimental period. It therefore seems justified to suggest that physiological signs of fatigue or maybe overreaching were present after the 6-week period with 10 matches. However, the analysis of training load clearly proved that the highest loads were applied during the last week compared with the previous weeks. These acute effects of the last week of competition could partly explain the observed physiological signs of fatigue at T2. This is supported by the distance covered during the YYIR1 that was reduced by 34.9%. A possible explanation for this decline could be impaired cell contractile properties. In fact, structural damage has been suggested to interfere with optimal muscle glycogen store replenishment which is required if the player performs prolonged high-intensity intermittent exercise [38]. The parameters of the repeated sprint schedule ability test (RSSA-test); RSSA_mean_ and RSSA_best_, increased by 2.6% and 3.8%, respectively. This performance enhancement may be explained by the observed 15.8% increase in lactate level measured at the end of the test. The increase in lactate can be explained by at least two hypotheses. First, it could be caused by an increase in energy production through the lactic anaerobic system and/or a decrease in energy production through the anaerobic alactic system. Accordingly, soccer players may exhibit decreases in stored phosphagen (ATP-CP) in the muscles at the end of an intense training period [40]. The second assumption is the decrease in the players’ ability to eliminate lactate as a result of the performance decline in the aerobic system [41]. We speculate that the decrease in performance in the first sprint during the RSSA-test is a result of the degradation of the efficacy of the alactic anaerobic system. The observed increase in time to complete the first sprint could be related to a depletion of the ATP-CP energy reserves [41]. This however is speculative and needs to be verified in future studies. Further, the examined decline in RSSA and YIYR1 performances may result from mechanical (e.g., force generation) and metabolic (e.g., glycogen depletion) impairments [42].

Soccer players’ SJ performance decreased by 2.5% while CMJ performance was unaffected. A potential explanation for this finding could be that alterations in peripheral neuromuscular function occurred which were induced by the reiteration of damaged skeletal muscle fibers [43]. These results confirm the fatigue state of elite soccer players after the congested period (10 matches over 6 weeks). However, it is important to note that in the current study, the assessment of physical fitness occurred 72 hours after termination of the congested period. Therefore, it appears plausible that playing multiple games over a prolonged period (6 weeks) together with an increased training load during the last week could have further reduced the time required to restore neuromuscular system properties. It is possible that changes in physical fitness were due to inadequate recovery during the congested match period, particularly during the last week of the intervention period. This may have caused the observed declines in protein synthesis rate, glycogen resynthesize, and the ability to generate force within the musculature [44], which would relate to muscular performance measures such as speed and power (RSSA_mean_, RSSA_best_, and SJ).

Similarly, Bangsbo et al. [45] suggested that the alteration of soccer players’ physical performance during the season might be due to the lack of in-week physical training caused by the mid-week matches. In this regard, Impelizzeri et al. [46] reported that soccer players’ ability to jump, sprint and perform repeated high intensity exercises was impaired after 6-weeks of training when playing two matches per week. Alternatively, reduced performance could be explained by increases in muscle soreness [47]. In general, muscle soreness peaks 24 to 48 hours after physical exercise. This exercise-induced phenomenon is called delayed onset muscle soreness (DOMS) [48].Furthermore, the deterioration of physical fitness may also be related to other psychological factors caused by the negative results of the team that won only 2 out of 10 games that were played during this period.

The present study does have a number of limitations that warrant discussion. First, we examined only one soccer team in this study. Hence, our sample size and the training regime of the subjects are limited. Second, only a small number of physiological and physical fitness tests were applied. Thus, our findings are specific to these outcomes only. Future studies should examine additional biochemical, immunological and psychological markers to further elucidate this research topic and provide more insight.

## Conclusions and practical implications

This study examined the effects of a congested match period on PV, hematological parameters and physical fitness in elite soccer players. The results of this study showed significant changes in various hematological parameters and physical fitness, which suggests potentially negative effects of the training load and match play during the experimental period. Specifically, the congested period of match play resulted in declines in physical fitness that seemed related to TWTL.

This research may provide useful information for coaches and medical staff with regards to TWTL and physical fitness performances during an intensive period of congested match play. Our findings may aid to better manage strain during exercise to avoid overreaching and overtraining, while reducing the risk of sustaining injuries in players. Our findings clearly illustrate the need for sufficiently long recovery periods at the end of the season. This is of particular importance for players who are nominated for their national teams and perform international tournaments after the regular season. Moreover, our results suggest that the recovery period after the end of the season should be individualized to take each player’s exposition in terms of match and training time into account. Finally, results from this study raise some concerns with regards to current practices in soccer clubs. Among the key questions are the application of team rotation and recovery strategies to reduce the risk of injuries. If the goal is to develop soccer players’ physical fitness over the course of a season, it is recommended to monitor physical and physiological demands during congested periods of match play.

## Supporting information

S1 Data Individual data of anthropometrics, hematological values and performances of the participating soccer players. (XLSX)

S2 Data Individual data of plasma volume variations of the participating soccer players. (XLSX)

S3 Data Individual data of training load, monotony and strain of the participating soccer players. (XLSX)
